# Using of Single-Layer Porcine Small Intestinal Submucosa in Urethroplasty on a Beagle Model

**DOI:** 10.1155/2022/1755886

**Published:** 2022-09-27

**Authors:** Daorui Qin, Xuejun Wang, Yu Mao, Yunman Tang

**Affiliations:** Department of Pediatric Surgery, Sichuan Academy of Medical Sciences Sichuan Provincial People's Hospital, Chengdu 610072, China

## Abstract

**Purpose:**

To evaluate the role of porcine small intestinal submucosa (SIS) in reducing fistula during urethroplasty and to observe its degradation process in beagle models.

**Methods:**

22 male beagles were divided into the SIS group and control group. All animals received surgical operation to establish the hypospadias model. Urethroplasty was followed. In the SIS group, the urethra was covered with a single-layer SIS material while no SIS material covered in the control group. At the 2^nd^, 4^th^, and 12^th^ weeks after the operation, there were 3, 3, and 5 animals in each group, respectively, sacrificed for surgical site histological examinations. The inflammation reaction and collagen hyperplasia levels were assessed. The fistula was identified by retrograde cystourethrography at the 4^th^ and 12^th^ weeks after the operation.

**Results:**

the incidence of urethral fistula was 25% (2/8) in the SIS group and 75% (6/8) in the control group. The inflammation reaction of SIS and control groups had no significant difference (*U* = 52.50, *P* = 0.58). The collagen fiber increased in both groups; however, the SIS group had a much more gentle increase compared to the control group (*U* = −0.00, *P* < 0.001). In the SIS group, the SIS material was roughly complete on the specimens 2 w after surgery but became loose and discontinuous 4 w after surgery and could not be found 12 w after surgery.

**Conclusion:**

The material can decrease the incidence of urethral fistula in the animal models, when used as a coverage layer. The SIS degradation process started 2 w–4 w after the operation and finished before 12 w in the animal model.

## 1. Background

Urethroplasty can be used in the treatment of urethral trauma, hypospadias, urethral stricture, urethral diverticulum, and other diseases. For a long time, urethral repair and reconstruction represented by hypospadias have been a great challenge for urologists due to the high incidence of postoperative complications. Urethral fistula is one of the most common complications of urethroplasty. Many efforts have been made to reduce urethral fistula after urethroplasty. Adding new urethral coverage is one of these efforts. Various autogenous tissue covering techniques have been widely reported. However, in these techniques, the extraction process of autologous tissue is time-consuming and difficult to master and the autologous tissue that can be used to cover may not always be sufficient, especially for patients who have experienced many failed urethral repair operations. Tissue engineering offers a silver lining to this dilemma. Porcine-derived small intestinal submucosa (SIS), one of the most typical tissue engineering material, is widely used in reconstructive surgery [1]. Several studies have reported the usage of SIS for urinary system reconstruction; however, few studies focused on the application of SIS on hypospadias repair [2, 3]. The limited research on SIS application in the repair of hypospadias was used as an onlay graft, and the results were depressing [4]. Herein, we reported the application of SIS as an additional coverage layer of the urethra during the hypospadias repair operation on a beagle model.

## 2. Materials and Methods

All animal procedures were approved by the ethics committee of our hospital and were performed according to the Chinese animal welfare regulation (GB/T1688.2), animal production license number: SCXK (Chuan) 2014-01, license number for the use of test animals: SYXK (Chuan) 2018-058.

The animals were raised in 800 mm × 900 mm × 830 mm metal cages with ambient temperature 20°C–26°C and relative humidity 40%–70%, with a 12 h light/dark cycle, and were allowed free access to food and water.

22 healthy male specific pathogen-free adult beagles were numbered and randomly divided into an observation group and a control group with 11 beagles in each group. The animals received different operations and numbered by the order that they received operations. All animals were given a single dose of cefovecin sodium (8 mg/kg, sc; Convenia®, Zoetis Japan Inc.) to prevent infection immediately after the operation. The urethral catheters were indwelled for 2 w unless they shed accidently.

The SIS materials were provided by Beijing Datsing Bio-Tech Co. Ltd., product specification: 6 cm × 1.5 cm, single layer. Before use, rehydration with normal saline was applied directly to the new urethra and the excess SIS material was cut off.

### 2.1. Operation

**Figure 1:**
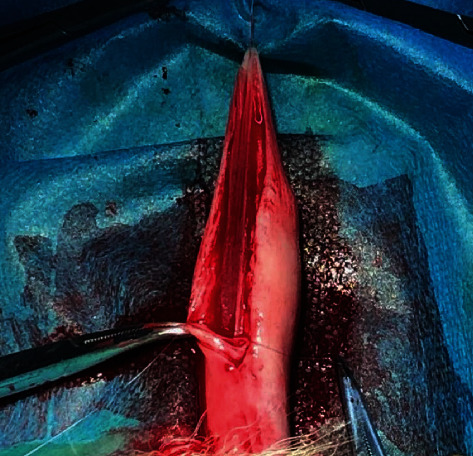
Surgical hypospadias animal model was established.

**Figure 2:**
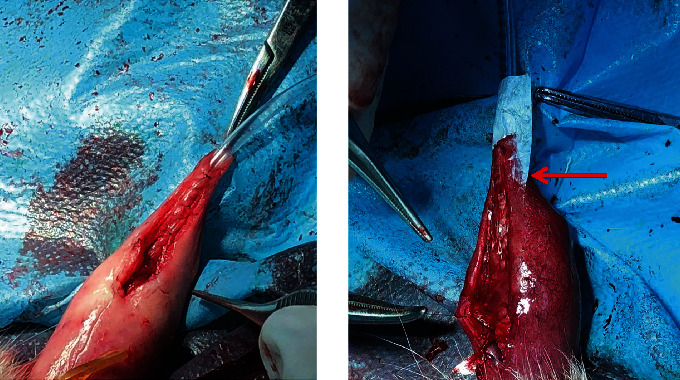
The SIS group animal's urethra was covered with the rehydrated SIS material and closed with an absorbable braided sutures.

The animals were anesthetized by an intravenously injection of 30 mg/kg pentobarbital sodium, and the abdominal skin was disinfected with povidone-iodine before the operation. The penis was degloved, and an 8F urethral catheter was inserted into the bladder and followed by tourniquet application to the base of penis. A 3–5 cm full-thickness incision was made in the ventral midline of the penis from the meatus to the base of the penis. Both sides of the corpus cavernous of the penis were sutured with an absorbable monofilament suture (PDS, 7-0, Ethicon Inc). The surgical hypospadias animal model was established (Figure 1). The repair operation was followed. During the reconstruction operation, both edges of the urethral plate were separated by about 1/3 and sutured with 7/0 PDS in a continuous running manner. The SIS group animal's urethra was covered with rehydrated SIS material and anchored to the repaired urethra. The skin was closed with an absorbable braided suture (VICRYL, 5-0, Ethicon Inc.) in an interrupted manner (Figure 2). The control group was covered with reconstructed urethra with the skin directly with an absorbable braided suture (VICRYL, 5-0, Ethicon Inc.).

### 2.2. Retrograde Cystourethrography

**Figure 3:**
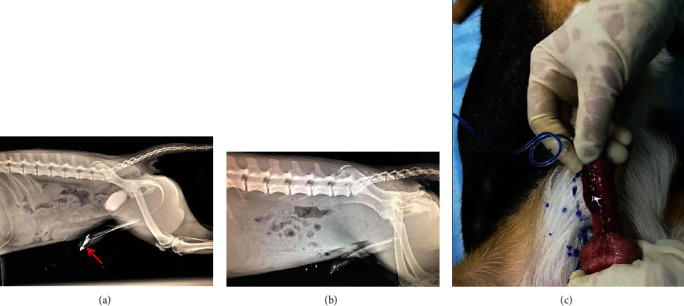
(a) Urethrogram demonstrates a urethral fistula and stricture (arrow). (b) A normal urethrogram. (c) The leakage of methylene blue solution diluted with normal saline from urethral cutaneous fistula confirms the presence of fistula.

The animal was anesthetized and the base of its penis was ligated with a tourniquet. Normal saline-diluted methylene blue injection (*V* : *V* = 25 : 1) was injected into the urethra through a 6F gastric tube with pressure. The leakage of blue fluid from the ventral side of the penis suggests a urethral fistula (Figure 2). After the fistula detection was completed, the animals were again catheterized and injected 1 : 1 diluted iodihydramol solution into the bladder to remove the catheter when a small amount of overflow occurred at the urethral orifice. The urological plain film was taken immediately, and urethral stricture and urethral fistula were recorded. (Figure 3).

### 2.3. Histological Observation

At 2 w, 4 w, and 12 w after operation, the animals were anesthetized and sacrificed. The baculum was removed in all penis specimens. All specimens were fixed with 10% formalin, then dehydrated, and embedded in paraffin and serial sectioned for histological observation. Hematoxylin-eosin (HE) and Mallory staining were performed. An experienced pathologist reviewed all the tissue sections and scaled the surgical site inflammatory reaction and collagen hyperplasia of the surgical site into four levels according to a quartering strategy adapted from Żywicka et al. [5] (Table 1).

### 2.4. Statistics

Data was analyzed using IBM SPSS Statistics version 25. Group differences in continuous variables were analyzed by Student's *t*-test or the Mann–Whitney *U*-test. Fisher's exact test was used for urethral fistula incidence rate and urethral stricture incidence rate. A *P* value < 0.05 denoted the presence of a statistically significant difference.

## 3. Results

All animals tolerated the surgical procedure well and survived for the whole postoperative time until being sacrificed. Redness and swelling of the surgical site were noticed in the early stage without special treatment and recovered within 4 weeks. Each group had 1 animal, had difficulty in retracting its penis properly after the operation, but recovered 2 w later without any intervention. The length of repaired urethra was 3.86 ± 0.69 cm in the SIS group and 3.85 ± 0.65 cm in the control group, with no statistical significance (*P* = 0.64, *t* = 0.19) (Table 2).

Urethrography indicated that 1/3 of the SIS group and 3/3 of the control group had urethral fistula at the surgical site 4 weeks after surgery. At 12 weeks after operation, urethral fistula was found in 1/5 of the SIS group and 3/5 of the control group. In total, the incidence of urethral fistula was 25% (2/8) in the SIS group and 75% (6/8) in the control group; the SIS material was effective in reducing the occurrence of urethral fistula (*P* = 0.04) (Table 2). Urethral stricture was found in 25% (2/8) of the animals in both two groups.

**Figure 4:**
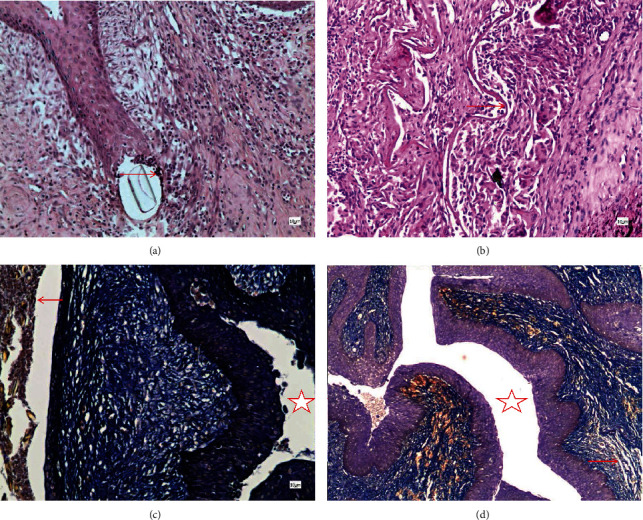
Surgical site urethra histological observation 2 w after operation. (a) Control, HE; (b) SIS, HE; (c) control, Mallory; (d) SIS, Mallory. The suture was found in (a) and (c), and acute inflammation cells were most observed around the suture (arrow). SIS material was identified in images (b) and (d) (arrow); there was no significant inflammatory cell proliferation around the material. The star marks the urethra in images (c) and (d).

**Figure 5:**
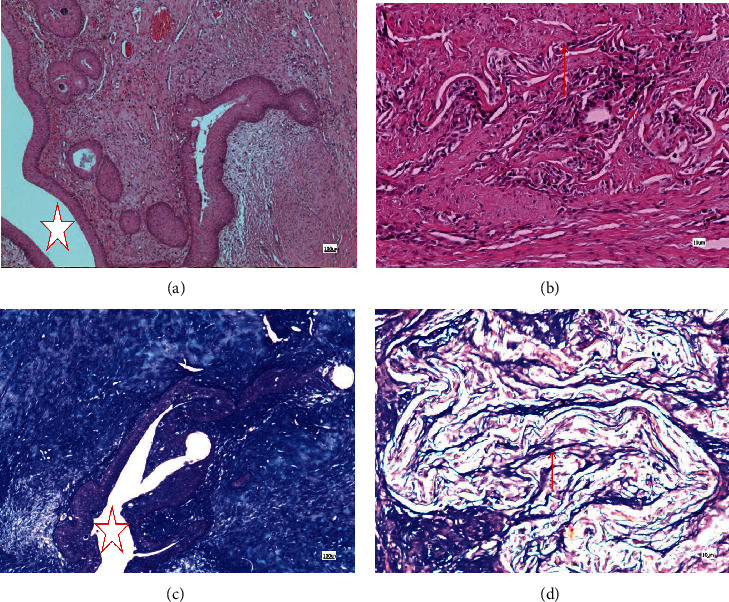
Surgical site urethra histological observation 4 w after operation. (a) Control, HE; (b) SIS, HE; (c) control, Mallory; (d) SIS, Mallory. The star marks the urethra in images (a) and (c). SIS material can noticed in images (c) and (d); the material partially disintegrates and the continuity is broken (arrow in image (b)). A small amount of blue-stained collagen along the material can be seen in image (d) (arrow). A large amount of blue-stained collagen can be observed in image (c).

**Figure 6:**
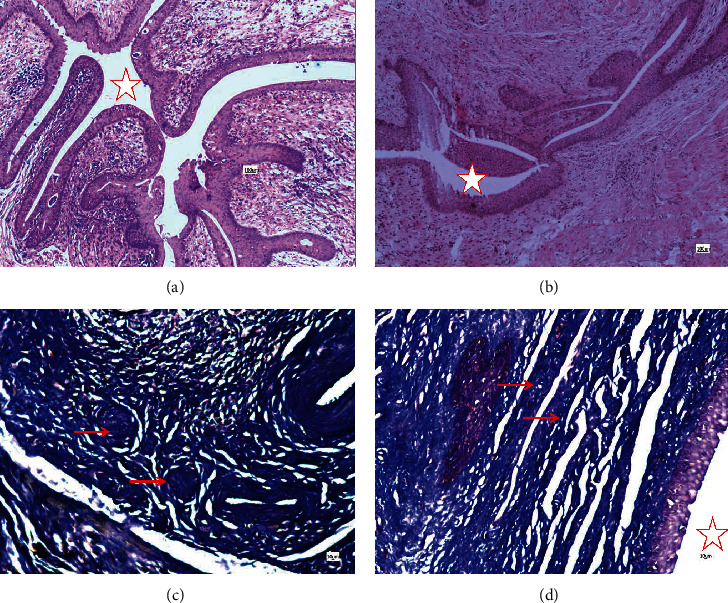
Surgical site urethra histological observation 12 w after operation. (a) Control, HE; (b) SIS, HE; (c) control, Mallory; (d) SIS, Mallory. The star marks the urethra in images (a), (b), and (d). A normal urethra was observed in images (a) and (b). SIS material was disintegrated and replaced by fibrous tissue. In image (c), a large number of hyperplastic and disordered collagen fibers can be seen, while in image (d), the original SIS material has been replaced by a regular arrangement of collagen.

Histological examination of the control group manifested a typical wound healing process. Mallory staining showed increased collagen fibers in the surgical area, and the boundary between the dense connective tissue and the cavernous body was not obvious, with collagen fibers interlaced and disordered. In the SIS group, acute inflammation cells were most observed around the suture at 2 w but no obvious inflammatory cells were observed in the material area (Figure 4). At 4 w, the material begins to degrade and a small amount of collagen begins to form along the material, while in the control group, a large number of disordered collagen fibers were found (Figure 5). At 12 w, a normal urethra was observed and the SIS material was disintegrated and replaced by fibrous tissue (Figure 6). The histological image review results were listed in table 3.The inflammation reaction was observed in both groups; however, there were no significant differences between the two groups (*U* = 52.50, *P* = 0.58) (Table 2). The collagen fiber increased in both groups, but the SIS group had a much more gentle increase compared to the control group (*U* = 0.00, *P* < 0.001) (Table 2).

## 4. Discussion

SIS is a natural extracellular matrix biomaterial, usually derived from the small intestine of swine. Currently, as well as commercially available, SIS can be used directly in clinical therapeutic activities. However, the application in urethral repair is rarely reported. SIS mainly consists of I and III type collagens, which contain FGF-2, TGF-beta, bFGF, and other growth factors [6, 7]. Because of containing these growth factors, SIS is thought to have a function to promote wound healing and reduce scar formation [8]. In addition, SIS also contains proteoglycan sulfate, vascular endothelial growth factor, and fibronectin, which could promote the adhesion and migration of endothelial cells, monocytes, and fibroblasts [6, 9]. It may also be for these reasons that SIS materials play a role in promoting wound healing and reducing urethral fistula during urethral repair. Orabi et al. [4] used a four-layer SIS material as an onlay graft in 12 hypospadias patients but failed in 3 patients with main complications due to graft infection. However, no serious infection was observed in our study. It seems that the SIS material is much more safer to be used as an additional coverage then be used as urethral substitute exposed to the urine directly. Instead of replacing a particular tissue, SIS materials provide a three-dimensional scaffold on which cells can grow and migrate. In this sense, more layers of SIS material coverage mean longer cell migration distances. Therefore, in urethral repair surgery, we prefer to use a single-layer SIS material rather than a multilayer cover.

As a biological product of heterogeneous origin, it is also worth noting whether it will cause rejection after implantation. In recent years, the presence of galactosyl (1,3) galactose (Gal) in porcine-derived SIS was supposed to account for the rejection response. However, a large number of clinical experiences and more than 1000 crossgermline transplantation experiments have confirmed that SIS has no immunogenicity and there is no response to directly stimulated immune experiments [10]. Another study found that natural antigalactose alpha1,3 galactose antibodies delay but do not prevent the acceptance of extracellular matrix xenografts [11]. In our study, no clear rejection reaction was also recorded and the inflammation reaction in the SIS group was almost the same as that in the control group, which was consistent with the results reported in the literature, again confirming the good biocompatibility of SIS materials [12].

Most fibers in the SIS are parallel to the long axis of the small intestine, and some fibers are staggered with the long axis of the small intestine. It gives SIS material good mechanical strength. Currently, SIS has been used in repair and reconstruction surgery [13–16]. The use of SIS materials in the urinary systems has also been reported [2–4, 16]. Kropp et al. [17] used SIS to repair the bladder of rats and found that the SIS was covered by transitional epithelium at the end of the 2^nd^ week, and normal bladder tissue was formed at the transplanted site 48 w later. In our study, at the end of the 2^nd^ week, only a few fibroblasts and collagen fibers were observed attaching to the SIS material; at the end of the 4^th^ week, the materials were partially degraded, and 12 w after the surgery, all materials degraded. It is difficult to distinguish the structure of the repair site from the normal structure. This difference in time consummation of material degradation may be due to the location of the material and the type of animal used. It may also be due to the fact that the migration rate of bladder transitional epithelium is different from that of fibroblasts. However, the SIS degradation time reported in our study is consistent with that reported by other literature [18]. SIS materials degrade during cell growth and migration and are eventually replaced by normal tissue. In our study, there was an obvious boundary between the cavernous body and the dense connective group, and the collagenous fibers were arranged regularly. It is much more close to the normal penis structure.

The main component of scars is collagen fibers. As a widely distributed tissue throughout the body, collagen fibers are involved in wound healing. However, excessive collagen proliferation can affect the appearance of the wound and may even cause local dysfunction. In this study, it was found that the proliferation of collagen fibers in the SIS group was significantly less than that in the control group. This suggests that the SIS material may inhibit scar proliferation to some extent. The reason may be that cells need certain extracellular scaffolds during tissue repair. The scaffolds include temporary scaffolds and permanent scaffolds. The temporary extracellular scaffold is mainly derived from the proliferation of cells in adjacent tissues. The SIS material acts as this “smart” temporary three-dimensional extracellular scaffold, providing cells to crawl in the early stage of the healing process and rapidly degrading after the initial healing to avoid excessive collagen proliferation in the wound.

Urethral stricture is another problem worthy of attention in urethral repair, as urethral stricture not only could bring pain to patients but also may lead to the failure of the whole operation. The main causes of urethral stricture are high tension at the operation site and tissue ischemia. This is one of the reasons why we tend to use single-layer SIS material for covering.

In this study, a model of hypospadias in beagles was established surgically. The beagle's penis size is comparable to that of humans and can provide a suitable penis length to mimic hypospadias in humans. However, because beagles do not have similar glans and coronal grooves as humans, the actual situation of hypospadias in humans cannot be completely simulated. To observe the materials' capability in preventing fistula, we designed a more demanding environment than the actual situation. In this study, the urethra of the control group was covered directly with the skin, but in practice, during most hypospadias operations, one or more autologous fascia is placed over the newly formed urethra to prevent fistula. This may also account for the high incidence of urethral fistula in this study. In addition, the incision is usually just healed within two weeks, which is rarely used to evaluate the surgical effect of hypospadias on clinical practice. Therefore, we did not include animals sacrificed 2 weeks after the operation when evaluating the occurrence of urethral fistula.

In conclusion, SIS materials have good biocompatibility. It did not change the normal healing of the urethral mucosa. The material can remodel the collagen fibers attached to the SIS material and grow regularly. It may potentially decrease the urethral scars in the beagle hypospadias model. The material can decrease the incidence of urethral fistula in the animal model, when used as a coverage layer. The SIS degradation process started 2 w–4 w after the operation and finished before 12 w in the animal model.

## Figures and Tables

**Table 1:** The histological evaluation criteria for inflammation and collagen hyperplasia.

Cell type	Score			
	1	2	3	4
Granulocytes	1–10^∗^	10–20^∗^	Heavy infiltrate	Packed
Macrophages	1–10^∗^	10–20^∗^	Heavy infiltrate	Packed
Lymphocytes	1–10^∗^	10–20^∗^	Heavy infiltrate	Packed
Collagen fiber	Mild	Moderate	Severe	Very severe

^∗^For large magnification ×400.

**Table 2:** The main statistic results of two groups.

	SIS group	Control group	*P* value
Repaired urethra length (cm)	3.85 ± 0.65	3.86 ± 0.69	0.64
Urethral fistula (*n*, %)	2, 25.00	6, 75.00	0.04
Urethral stricture (*n*, %)	2, 25.00	2, 25.00	1.00
Inflammation reaction score	2.91 ± 0.31	3.18 ± 0.23	0.58
Collagen fiber score	1.27 ± 0.14	3.18 ± 0.12	0.000

**Table 3:** Histological image review results of two groups.

Animal number	Group	Harvest time	Fistula (Y = yes, N = no)	Inflammation reaction score	Collagen fiber score
S1	SIS	12w	N	2	1
S2	SIS	12w	N	1	1
S3	SIS	12w	Y	3	1
S4	SIS	12w	N	2	1
S5	SIS	12w	N	2	1
S6	SIS	4w	N	3	2
S7	SIS	4w	N	3	2
S8	SIS	4w	Y	4	2
S9	SIS	2w	Undo	4	1
S10	SIS	2w	Undo	4	1
S11	SIS	2w	Undo	4	1
C1	Control	12w	N	2	3
C2	Control	12w	N	2	3
C3	Control	12w	Y	3	3
C4	Control	12w	Y	3	3
C5	Control	12w	Y	3	3
C6	Control	4w	Y	3	3
C7	Control	4w	Y	3	3
C8	Control	4w	Y	4	3
C9	Control	2w	Undo	4	4
C10	Control	2w	Undo	4	4
C11	Control	2w	Undo	4	3

## Data Availability

Data are available upon request.
